# Psychiatric consultations in cardiac surgery, vascular surgery and transplant units: an eight-year retrospective study

**DOI:** 10.1192/j.eurpsy.2026.12020

**Published:** 2026-07-31

**Authors:** S. Florek, A. Suchodolski, M. Szulik, T. Hrapkowicz, R. Pudlo

**Affiliations:** 1 Department of Psychoprophylaxis, Faculty of Medical Sciences in Zabrze, Medical University of Silesia, Tarnowskie Góry; 2 Department of Cardiology and Electrotherapy, Silesian Centre for Heart Diseases; 3 Department of Cardiac, Vascular and Endovascular Surgery and Transplantology, Silesian Center for Heart Diseases, Zabrze; 4 Department of Psychoprophylaxis, Faculty of Medical Sciences in Zabrze, Medical University of Silesia, Tarnowskie Góry, Poland

## Abstract

**Introduction:**

Psychiatric comorbidity is increasingly recognised as a determinant of outcomes in patients undergoing major cardiac or vascular surgery and in transplant recipients. Depression, anxiety, adjustment disorders and delirium can complicate perioperative courses, prolong hospitalisation and adversely influence adherence to treatment. Despite these risks, systematic data on psychiatric consultations in these settings remain sparse.

**Objectives:**

To characterise the frequency, demographic profile, clinical indications and therapeutic consequences of psychiatric consultations performed in cardiac surgery, vascular surgery and transplant wards of a tertiary heart centre over an eight-year period.

**Methods:**

A retrospective review of consultation records (January 2016–March 2024) at the Silesian Centre for Heart Diseases (Zabrze, Poland) was undertaken. From 2,196 consultations retrieved, 720 adult in-patients met inclusion criteria (complete data; consultation during admission to cardiac surgery, vascular surgery or transplant departments). Variables included sex, age, primary medical diagnosis, prior and newly initiated psychopharmacotherapy, and consultations addressing capacity or other legal/ethical issues. Descriptive statistics and Spearman rank correlations were applied.

**Results:**

Of 720 consultations, 512 concerned men (71.1%) and 208 women (28.9%); mean age was 53.2 ± 14.5 years. The majority were undertaken in transplant units (n = 579, 80.4%). New psychopharmacological treatment was advised in 30.9% of cases, with highest rates among patients with respiratory disorders and heart failure. Most referrals involved middle-aged adults (36–65 years). Young adults (18–25 years) showed the greatest proportion requiring de novo therapy. Legal/ethical assessments and recommendations for psychiatric re-evaluation within four weeks were most frequent in patients with heart failure and in those aged >35 years. Correlations between age and other variables were uniformly weak, except for a slight positive association with the need for pharmacotherapy.

**Conclusions:**

Psychiatric disorders are prevalent among patients hospitalised for cardiac or vascular surgery and especially among transplant candidates or recipients. Nearly one third of consultations resulted in initiation of psychotropic medication, underscoring the magnitude of unmet mental health needs in these populations. The predominance of referrals from transplant wards reflects the psychological burden of organ failure and transplantation. Early liaison and systematic screening—particularly in younger adults and those with respiratory or heart failure diagnoses—may facilitate timely treatment, reduce perioperative complications and improve adherence after discharge. Close cooperation between surgical, transplant and psychiatric teams is essential to optimise outcomes.

**Disclosure of Interest:**

None Declared

